# Process evaluation of comprehensive sexuality education programme in Zambia: a focus on contextual factors, mechanisms of impact, quality of development and implementation process

**DOI:** 10.1186/s12913-024-11083-z

**Published:** 2024-07-25

**Authors:** Bright Mukanga, Siyabonga Blessing Dlamini, Myra Taylor

**Affiliations:** 1 Discipline of Public Health Medicine, School of Nursing and Public Health, University of KwaZulu-Natal, Glenwood, Durban, 4041 South Africa; 2 Michael Chilufya Sata School of Medicine, Public Health Department, Copperbelt University, PO BOX 71769, Ndola, Zambia; 3 Cancer & Infectious Diseases Epidemiology Research Unit, College of Health Sciences, University of KwaZulu-Natal, Durban, South Africa

**Keywords:** Adolescents, Process evaluation, School, Comprehensive sexuality education, Zambia

## Abstract

**Background:**

Comprehensive sexuality education (CSE) is critical in addressing negative sexual and reproductive health (SRH) outcomes among adolescents. Yet in many low- and middle-income countries (LMICs) including Zambia, little is known about the impact, realities of CSE implementation, the quality of teaching and the comprehensiveness of the content covered.

**Methods:**

Our approach was informed by a process evaluation incorporating recommendations by the European Expert Group guidance on evaluating sexuality education programmes and the Medical Research Council (MRC) guidelines on process evaluation. The development process and quality of CSE implementation were assessed using eight and six quality criteria respectively. In-depth interviews (IDIs), focus group discussions (FGDs), document analysis and classroom observation were employed to assess contextual factors, implementation process and mechanisms of impact of CSE. In-depth interviews (50) and focus group discussions (2) with seven pupils in each group were conducted among 64 purposefully selected participants. The sample comprised pupils (35), parents (4) and teachers (17) from nine secondary schools (four peri-urban, four urban and one rural), policymakers (4), and religious leaders (4). We employed deductive content analysis to analyse the data.

**Results:**

Contextual factors that influenced the implementation of CSE included: (1) piecemeal funding for the CSE programme; (2) lack of monitoring programmes in schools; (3) lack of community engagement; (4) religious and socio-cultural barriers; (5) lack of skills and competency to teach CSE; and (6) insufficient time allocation for CSE. The assessment of the quality of the development of CSE revealed: (1) a lack of sexual diversity; (2) no meaningful participation of pupils in programme implementation; (3) a lack of stakeholder engagement during programme implementation; (4)  lack of gender sensitivity; and (5) lack of human rights approach. Assessment of the quality of the implementation of CSE revealed: (1) no evidence of skill-based CSE teaching; (2) no linkage between CSE and SRH services in the communities; and (3) a lack of incorporation of multiple delivery methods during CSE teaching. The mechanisms of impact of CSE were related to the acceptability and positive changes in pupils’ SRH practices.

**Conclusion:**

The complex influences of contextual factors during CSE implementation highlight the need for contextual analysis during the interventional design. Co-creation of the CSE programme through stakeholder participation could reduce social opposition and enable a culturally sensitive CSE. Comprehensive teacher training, a guiding curriculum as well as setting of appropriate monitoring tools and indicators are likely to enhance the quality of CSE implementation.

## Background

School-based comprehensive sexuality education (CSE) can enable adolescents to realise their sexual and reproductive health (SRH) [1–5]. SRH entails physical and emotional well-being which incorporates the ability to be free from unwanted pregnancy, unsafe abortion, sexually transmitted infections (STIs) including human immunodeficiency virus (HIV) and all forms of sexual violence and coercion [6]. This is enshrined in the goals set at the 1994 International Conference on Population and Development programme which highlighted the role of governments in promoting adolescents’ SRH through delivering school-based sexuality education [7, 8], and the Agenda 2030 on enhancing universal health coverage for all [9–11]. Adolescents represent a growing number of people living with HIV globally. Estimates indicate that in 2019, about 2.4 million adolescents aged between 10 and 19 were living with HIV, with about 170,000 (53,000-340,000) infected with HIV in 2019 [12]. The high prevalence of HIV among adolescents is compounded by vulnerabilities in the socio-ecological environment in which adolescents live and grow [13, 14]. The transition from adolescence to adulthood is marked by physical, emotional, cognitive and psychological changes which increase adolescents’ vulnerabilities to risky sexual behaviours such as multiple sexual partners [15–17].

Comprehensive sexuality education is an “age-appropriate, culturally relevant approach to teaching about sexuality and relationships by providing scientifically, accurate, realistic and non-judgement information” [18]. Comprehensive sexuality education incorporates cognitive, emotional, physical and social aspects of sexuality and emphasises acquiring life skills to enable adolescents to make informed sexual health decisions [8]. Several benefits have been associated with CSE implementation, such as delayed onset of sexual activity and reduction of risky sexual behaviours such as unwanted pregnancies and multiple sexual partners [19]. Comprehensive sexuality education can also enable adolescents to acquire necessary information and skills regarding their bodies’ function and can demystify wrong sexual and cultural notions [9, 20].

However, in many settings, implementing CSE in schools has been patchy and has faced social and religious opposition from stakeholders such as parents, religious leaders, and other civil society organisations [21, 1]. Chavula et al. [9], report that deep-seated discomfort about adolescent sexuality persists among stakeholders because of social, cultural, religious and structural factors. These socio-cultural barriers are reinforced by laws and policies that regard activities such as condom promotion among adolescents as being contrary to local social norms [22]. As such, CSE teaching mainly takes the abstinence approach with a weak focus on gender and human rights, with topics such as homosexuality and abortion invariably excluded [23]. Furthermore, restrictive cultural and policy environments hinder adolescents’ access to much-needed SRH services [9, 24].

To support effective CSE implementation, assessment of contextual factors and quality of implementation is crucial [8, 25, 26]. Multiple studies have stressed the need for effective evaluation of CSE to cultivate robust programme outputs [25, 27–30]. Process evaluation has been employed in many interventions to assess the contextual factors and causal pathways [31, 32, 30]. This is especially critical for complex interventions such as CSE that consist of different components and dynamic interactions in the socio-cultural context [33, 34]. The MRC guidelines provide a framework for addressing contextual factors influencing CSE implementation during the development and implementation of CSE [25, 30]. These guidelines highlight the relationships among the context, implementation, and mechanisms of change and how these enhance the quality and effectiveness of sexual health interventions [8, 27]. The European Expert Group guidance on the evaluation of holistic sexuality education has been used to assess the quality of implementation and developing process of CSE [30]. It provides appropriate indicators for assessing the quality of the development and implementation process of CSE through eight and six assessment criteria respectively [35]. However, despite many low and middle-income countries (LMICs) having implemented CSE curricula, gaps still exist in CSE programme implementation and evaluation [1].

Zambia is one of the countries with the highest prevalence of HIV in the world [36], with a reported HIV national prevalence of 11% among adolescents and adults aged 15 years and above. This has a huge bearing on the socio-economic development of the country because adolescents constitute 24% of the total population of Zambia [37]. To reduce adolescent risky sexual behaviours such as early marriages, unintended pregnancies, unsafe abortions, and gender-based violence, the government introduced policies such as the Education Act of 2011, the Adolescent Health Strategic Plan [9], and the 2017 test-and-treat-all policy or “90-90-90 strategy” [38]. Further, to scale up access to SRH and knowledge among school-going adolescents, the Reproductive Health Act was replaced by a new CSE framework (2014) which includes themes such as gender, relations, information on contraceptives, attitudes, and values [39, 40]. The 2014 CSE framework is centred on supporting adolescents in delaying their sexual debut, reducing sexual partners and unplanned pregnancies and increasing safer sexual practices [3]. As such, it was integrated into carrier subjects in all schools in 2014 and targets children and adolescents from grades 5 to 12 [9, 39].

Despite these policy gains, the content of the new CSE framework remains contested due to fears relating to disrupting social and cultural norms, and teachers tend to alter the CSE lessons and hold back content perceived to be problematic and in conflict with local norms [3, 41]. In the development and implementation process of the CSE framework, key stakeholders such as religious leaders, civic leaders, parents, and youths were left out [39]. Additionally, adolescents have limited access to CSE, and the implementation process has been patchy [42]. Estimates indicate that from 2017 to 2018, a total of 28,669 girls became pregnant and only 49% of these pregnant primary school girls returned to school in 2019 [43]. Studies on CSE in Zambia have mainly focussed on barriers and facilitators to the implementation of CSE [40, 44]. However, little is known about the impact, realities of CSE implementation, the quality of teaching and the comprehensiveness of the content covered [3, 1, 9]. We conducted this process evaluation to assess the development process of CSE, the quality of the implementation, the mechanisms of change and contextual factors influencing CSE implementation.

## Methods

### Study sites

The study was conducted in nine purposefully selected public secondary schools in Kitwe district of the Copperbelt province, Zambia. The province has a population of 2,669,635 as of 2020 [45], and the third-highest HIV prevalence nationally of 14.2% [46]. Kitwe district has a population of 762,950 as of 2022 and has many socio-economic activities, including mining, farming, and small business enterprises [47]. Social and structural vulnerabilities exist in the district and contribute to the increase in HIV acquisition and act as barriers to accessing SRH services among key populations such as adolescent girls and young women (AGYW) [48]. Legal and restrictive cultural contexts in the district contribute to stigma and discrimination against adolescents’ access to SRH services [49]. Kitwe district has 16 public secondary schools and 17 private secondary schools. The schools were selected to include different geographic areas (four urban, four peri-urban, and one rural) and all had experience of implementing the CSE programme. All selected schools permitted us to conduct the study.

### Sampling and target population

We employed criterion-i purposeful sampling to select participants comprising 35 grade 12 pupils aged between 16 and 20 years, 17 teachers, 4 parents, 4 policymakers, and 4 religious leaders. Teachers and pupils were selected from the nine secondary schools that had implemented the CSE programme. According to Palinkas et al. [50], criterion sampling is a type of purposeful sampling where information-rich participants who meet predetermined criterion are selected. As such, we purposefully selected participants to include key informants with diverse views and experiences about CSE, with grade 12 teachers selected across different subjects, grade 12 pupils selected across different classes who were members of school-based sexual health clubs, and parents and religious leaders who were part of the parent-teacher associations in different schools.

### Inclusion and exclusion criteria

We included grade 12 pupils who had been learning CSE from grade eight to twelve. In addition, we included policymakers and teachers who were involved in the implementation process of CSE. Grade eight to eleven pupils were excluded from the study.

### Study design

We employed a process evaluation that incorporated recommendations by the MRC on process evaluation [25] and the European expert group guidance on the evaluation of holistic sexuality education programmes [35]. The MRC provided guidelines for assessing contextual factors, implementation processes (fidelity, dose, each) and mechanisms of impact [25]. As such, we developed the interview guides using MRC constructs. The European Expert Group guidance on the evaluation of sexuality education assesses the quality of the development and implementation process of CSE and highlights appropriate indicators and methods for evaluating any sexual health intervention [35]. This is a eight and six-criteria assessment of the quality of the development and implementation processes respectively. Key indicators of the European Expert Group guidance on the evaluation of sexuality education programmes include: age-appropriateness; gender sensitivity of sexuality education; pupil involvement; sexuality education training and skills; and interactive teaching. Naturalistic inquiry through in-depth interviews (IDIs) and focus group discussions (FGDs) enabled a thick description of teachers’, policymakers’, pupils,’ parents', and religious leaders’ experiences of the CSE programme in their natural settings.

### Data collection

We employed a validated quality criteria assessment developed by the European Expert Group Consensus agreement to assess the quality of the development and implementation process of the CSE programme [30, 35]. Data on contextual factors and implementation process (fidelity, dose, each) were obtained through IDIs and FGDs. Classroom observation was done at nine secondary schools. Furthermore, we conducted a document analysis of the Zambian CSE framework and teacher manual. Using the MRC framework, we assessed fidelity (whether CSE lessons were being delivered as per the curriculum and international guidelines), reach (how many pupils attended CSE lessons) and dose (whether CSE lessons were delivered in the planned time duration).

### Quality of the CSE development process

We assessed the quality of the development process  of CSE using the eight-criteria assessment, which included: ‘a positive approach to sexuality’, ‘age appropriateness’, ‘gender sensitivity’, ‘comprehensiveness’, ‘pupil involvement’, ‘cultural and socially responsive, human rights approach’, and the ‘quality of the educator manual’. This criteria assessment was led by the main author and was verified by all members of the research team.

### Quality of CSE implementation

The six-criteria assessment included: ‘CSE educator training’, ‘completeness of the CSE curriculum’, ‘use of multiple methods’, ‘obligatory programme’, ‘linkages with sexual and wellbeing services’, and ‘ensuring a convenient group atmosphere for adolescents to express themselves freely’. The responses to these criteria were a three-point Likert scale where ‘0’ indicated that the criteria were not addressed, + indicated partially addressed criteria and + + indicated a fully addressed criterion [30, 35].

### Contextual factors, implementation process and mechanisms of impact

The IDI and FGD guides were developed using the MRC constructs and assessed contextual factors, implementation process and mechanisms of impact of CSE [25]. Fifty (50) IDIs were carried out and included 4 parents, 17 teachers, 4 policymakers, 21 pupils, and 4 religious leaders (see Table 1). Additionally, 14 pupils participated in two FGDs, each group had seven pupils. One FGD group was mixed-sex and the other group was same-sex (girls only). FGDs among pupils were carried out to leverage the synergistic effect of group discussions, direct personal interactions and ways in which individuals are influenced by others in a group situation. We adapted the IDI and FGD guides that were used in the Kemigisha et al. studies [8, 30]. Data collection lasted 4 months, from January to April 2023. We engaged research assistants trained in qualitative research who were familiar with the local language (Bemba). Interviews and FGDs were held in secluded places away from the administration blocks but within the school premises to ensure privacy and confidentiality. Before starting the interviews, permission was obtained from the participants to record the interviews. Data collection continued until theoretical saturation was achieved,this was when no new insights emerged from the IDIs and FGDs. The IDIs lasted between 40 and 50 min, and the FGDs for 60 min.

**Table 1:** Number of IDIs and FGDs for different stakeholders

Stakeholders	Number of In-depth interviews	Number of FGDs
Pupils	21	2
Teachers	17	0
Parents	4	0
Religious leaders	4	0
Policymakers	4	0
**Total**	**50**	**2**

### Document review

We further conducted a document review of the Zambian sexuality education framework and CSE teacher’s guide to assess whether it conformed to the CSE international guidelines. Additionally, a document analysis assessed the content of the Zambian CSE framework and the training records for teachers in all nine schools. We used the UNESCO international guidelines on sexuality education to assess age appropriateness and the quality of the education manual [51].

### Classroom observation

We conducted a total of nine classroom observations within three weeks at nine secondary schools to triangulate findings from IDIs and FGDs. The observation was done without interrupting the lessons. A standard form was used to record what was happening in the classroom. Classroom observation was particularly done to identify aspects of CSE that teachers emphasised, time allocated to teaching CSE, pupils’ participation and whether multiple methods were implemented during the lessons. Data from observations were coded thematically.

### Process evaluation research questions

The process evaluation research questions were guided by recommendations by MRC guidelines on process evaluation. The questions were centred on CSE programme development, context, implementation of CSE and mechanisms of impact (Table 2).

**Table 2:** Process evaluation research questions.  Adapted from [30]

Process evaluation domain	Research Question	Document Analysis	Qualitative data
Programme development	Did the intervention align with internationally recognized quality criteria for sex education?	**x**	
Context	What were the contextual factors that may have affected the intervention implementation and outcomes?	**x**	**x**
Implementation of the intervention	Did the implementation align with internationally recognised quality criteria for sex education?	**x**	**x**
Mechanisms of impact	What were participant responses to and interactions with the intervention?		**x**
	To what extent was the intervention accepted by stakeholders? (Pupils, teachers, parents)	**x**	**x**

### Data analysis

Data from the quality assessments of the development and implementation process of CSE were summarised. We employed validated scoring criteria developed by Evaluation of Holistic Sexuality Education (HSE): A European Expert Group Consensus Agreement (2016) [35]. The scoring criteria was a three-point likert scale where “0” indicated the criterion not addressed,+ for partially addressed and + + for fully addressed. As such, a total of eight criteria were considered for quality assessment  for CSE development and included: having a positive approach to sexuality, age appropriateness, gender sensitivity and so on (Table 4). Additionally, a total of six criteria were considered for the quality of implementation and included: educator training, completeness of curriculum, use of multiple methods and so on (Table 5).

Observation data were coded thematically. We further listened to audio recordings from the IDIs and FGDs, after which we transcribed them verbatim in the English language. A language translator was engaged to translate Bemba transcripts into English and back-translated them into Bemba to enhance translation accuracy. We reread the participant manuscripts to note any similarities between and within the participants’ accounts. We employed deductive content analysis using predetermined constructs from the MRC framework which included: contextual factors; implementation process (fidelity, dose, reach) and mechanisms of impact. The themes and subthemes were then generated manually. We employed five steps of deductive content analysis recommended by Bingham [52] as follows:

i. Organising data
ii. Sorting data into relevant topical categories
iii. Open/initial coding
iv. Identifying patterns, themes, and findings
v. Applying theory and explaining findings

### Ethical considerations

Approval for the study was granted by the Tropical Diseases Research Centre in Ndola, Zambia (IRB Registration Number: 00002911 & FWA Number: 00003729) and permission was granted by the National Health Research Authority Zambia (Ref No: NHRA000001/01/06/2022) and the Biomedical Research Committee of the University of KwaZulu-Natal in Durban, South Africa approved the study (Approval number: BRE/00004141/2022). Written informed consent was obtained from all participants before data collection. We followed the standard procedures of informed consent and ethical research conduct, which included anonymity and confidentiality. Before the actual data collection, letters of support were obtained from the District Education Office and all the participating schools. For adolescents under the age of 18 years, we obtained assent from their parents and guardians to participate in the study. Participants were informed about the purpose, benefits, and possible harms of the study. We anonymised all data records and ensured privacy and confidentiality.

### Trustworthiness and rigour of the study

We employed Guba and Lincoln’s criteria of credibility, dependability, transferability, and confirmability to enhance the study’s trustworthiness [53]. Credibility in our study was ensured through prolonged engagement with all the participants. We spent four months understanding the natural settings of participants. This helped in building trust and rapport with participants and exploring their experiences of the CSE programme. Through peer debriefing, we had our field notes validated and critiqued by research assistants who had degree qualifications in demography and social work and had experience in qualitative data analysis and collection. Multiple methods of data sources such as document analysis, participant observation, IDIs and FGDs were employed to enhance the credibility of the findings [54]. Thick description was enhanced through robust data collection until the saturation point. Participant recruitment in the study continued until data saturation was reached, this increased the scope, adequacy, and appropriateness of the data. We engaged independent qualitative researchers to validate our interview and focus group transcripts. The independent researchers were public health experts who specialised in qualitative research audits. This was key in ensuring the dependability of our data. Additionally, we engaged an independent qualitative research expert to conduct an audit trail of the whole research process, from data collection to transcription and analysis which was critical to enhancing the confirmability of our study findings.

## Results

Sixty-four subjects (64) participated in interviews and focus groups (Table 3). This included 35 pupils (19 boys, 16 girls) mean age (SD) 17.5 (0.95) and 17 teachers (11 females, 6 males) mean age (SD) 38.8 (7.3) across nine schools, four parents (two females, two males), four policymakers (two females, two males) and four religious leaders (two females, two males).

**Table 3:** Participant characteristics

Study Sample	Characteristic	Number of participants (*n*(%))
IDIs		
Pupils (*n* = 35)	Female	19 (54.3)
	Male	16 (45.7)
Mean age (SD)		17.5 (0.95)
Teachers (*n* = 17)	Female	11 (64.7)
	Male	6 (35.3)
Mean age (SD)		38.8 (7.3)
School (*n* = 9)	Peri-urban	4 (44.4)
	Urban	4 (44.4)
	Rural	1 (11.1)
Parents (*n* = 4)	Female	2 (50.0)
	Male	2 (50,0)
Policymakers (*n* = 4)	Male	2 (50.0)
	Female	2 (50.0)
Religious leaders (*n* = 4)	Male	2 (50.0)
	Female	2 (50.0)

### Assessment of the development and implementation quality of the CSE programme

Here, we present results on the assessment of the development and implementation quality criteria using recommendations by the European Expert Group Consensus Agreement on the evaluation of holistic sexuality education programmes.

### Assessment of the development quality criteria

Assessment of the quality of the development process of CSE revealed that four out of eight were partially addressed, and these included a positive approach to sexuality, gender sensitivity, comprehensiveness, and human rights approach. Two were not addressed and included pupil involvement and cultural and social responsiveness. Only two were fully addressed and included age appropriateness and quality educator manual (Table 4).

**Table 4:** Quality criteria of the development process of the CSE programme in Kitwe district. Ketting et al. [35]

Quality criteria	Evidence	Quality assessment category: 0 = not addressed, +=partly addressed, ++=fully addressed
Positive approach to sexuality	Document analysis showed that the CSE framework had a positive approach in terms of tone, illustrations, and examples of positive sexuality such as acceptance of homosexuality, but the implementation lacked all these elements.	+
Age appropriateness	Document analysis showed that the CSE framework had increased thematic depth with increasing age from grade 5 to grade 12.	++
Gender sensitivity	Classroom observation showed that the CSE lesson delivery lacked gender sensitivity and appropriate vocabulary when referring to sexual identities.	+
Comprehensiveness	Document analysis and classroom observation showed that insufficient detail of the CSE topics was covered	+
Pupil involvement	Classroom observation and document analysis showed that no mechanisms were in place for the meaningful participation of pupils in programme development and implementation.	0
Human rights approach	Document analysis showed that the programme included social values with an emphasis on children’s rights but did not include discrimination due to sexual diversity.	+
Cultural and socially responsive	Interviews showed no evidence of the formation of community boards to advise on the content of CSE and no stakeholder engagement during the planning and implementation phases.	0
Quality educator manual	Document analysis showed that the CSE training manuals had learning outcomes and described content and delivery modes adequately	++

### Assessment of implementation quality criteria

Assessment of the quality of implementation of CSE revealed that only one out of the six quality criteria of the implementation of CSE were fully addressed, which included obligatory programmes. Four of the criteria were partially addressed and one criterion was not addressed at all and included linkage with relevant SRH services (Table 5). The document analysis showed that the obligatory programme was fully addressed because the CSE programme was obligatory for all the pupils in the nine schools. In-depth interviews and document analysis showed a lack of extensive training in CSE among teachers and a lack of materials and support for teachers which compromised the fidelity of the programme. Also, there was insufficient time allocation for CSE at the school level. Most teachers only allocated 3–5 min to teaching CSE which affected the dose and depth of CSE lessons. Furthermore, document analysis revealed that the CSE programme was not linked to any existing SRH services in the community.

**Table 5:** Quality assessment of CSE implementation in Kitwe district. Ketting et al. [35],

Quality criteria	Evidence	Quality assessment category: 0 = not addressed, +=partly addressed, ++=fully addressed
Educator training/skills	Training records indicated that most teachers had not received training in CSE except guidance teachers.	+
Completeness in curriculum delivery	Qualitative data showed insufficient time allocation since CSE was integrated into other carrier subjects and teachers could allocate only five minutes which showed a lack of in-depth delivery of CSE in most schools.	+
Multiple methods used	Data from in-depth interviews and document analysis showed that few teachers incorporated multiple methods and CSE activities lacked activities to enhance skills and values among pupils.	+
Obligatory program	Document analysis showed that school teachers mobilised all the pupils within target classes for the program and attendance lists showed regular participation.	++
Ensuring a convenient group atmosphere for adolescents to express themselves freely	Document analysis, IDIs and FGDs showed that only two out of nine schools had youth-friendly corners and pupils had the opportunity to express themselves on issues of sexuality. However, during the CSE, most pupils did not participate due to limited time.	+
Linkages with relevant sexual and well-being services	Interviews and document analysis showed that the CSE programme was not linked to any SRH services in the community health facilities.	0

### Implementation of the CSE programme

Through IDIs, FGDs, document analysis, and classroom observation, we assessed contextual factors, implementation process (fidelity, dose, reach) and the mechanisms of impact using the MRC guidelines on process evaluation.

### Contextual factors

#### Religious and socio-cultural barriers

Implementation of CSE faced social opposition from stakeholders such as religious leaders and some teachers because of perceived conflict with religious and cultural norms. The restrictive cultural context on acceptance of some topics on sexual behaviour and diversity influenced the implementation of CSE in that most teachers held back or skipped culturally sensitive topics. IDIs and FGDs revealed that teachers had difficulty in reconciling personal religious and cultural beliefs and some of the content of the CSE.

*My religious and cultural beliefs do not permit me to teach such things [sex before marriage and using contraception] to adolescents. As a Christian, sex is meant for people who are married only, so I find it very difficult to teach* (Teacher, Female 35yrs, IDI, School 6).

*I normally choose what to teach and I skip content that conflicts with my religious beliefs. I know we are required to teach everything, but my conscious cannot allow me to teach things that are against my religious beliefs* (Teacher, Female, 36, IDI, School 7).

Additionally, some pupils expressed concern that some teachers were too shy to teach CSE, especially on culturally sensitive topics.

*Some teachers do not teach CSE because they say that those things are supposed to be taught when a girl or a boy is entering into marriage, and those who teach feel shy to talk about sexual issues, especially some male teachers* (Pupil, Female,18yrs, FGD 1, School 3).

There was a negative perception of the CSE programme in the media with the programme facing backlash among key stakeholders such as religious leaders and civic leaders. The implementation process was further hampered by the political pushback due to a restrictive policy landscape where the age of consent to sexual intercourse was 16 years and same-sex relationships were criminalised.

*As a country, we don’t believe in the promotion of homosexual rights. We have joined hands with lawmakers who have called for the revision of the sexuality framework in line with Zambian cultural and Christian values (Religious leader 1, Male 44 yrs, IDI).*

#### Lack of community engagement

Qualitative data showed that the implementation of CSE lacked a participatory approach to engage stakeholders in the design and implementation of CSE. Specifically, religious leaders, pupils and parents cited a lack of engagement during the planning and implementation of CSE.

*I think they didn’t do a good job in engaging stakeholders such as teachers, parents, pupils, and religious leaders during the development and implementation of the programme. It has come with negative opinions from the public, who are the parents, and some teachers have no idea what the program is all about. And then finally to the learners, I feel the learners have misunderstood the programme* (Parent1, Female 49yrs, IDI).

#### Piecemeal funding for the CSE programme

Stakeholders (teachers, and policymakers) expressed concern about the lack of financial support from the central government evidenced by the lack of teacher training and CSE teaching materials and manuals. Invariably, funding of CSE was done by partners and donors such as the USA Peace Corps, which was usually fragmented.

*We are unable to implement the programme effectively in all schools due to lack of funding. The teacher training and all the funding was done by the Peacecorps organisation, and since then, we have not had financial support from the government.* (Policymaker1, Female, 42 yrs, IDI).

#### Lack of monitoring programmes in schools

Monitoring and evaluation form an essential component of an effective process evaluation as it enhances effective CSE programme output and fidelity [25]. This is also important in ensuring that the implementation is done as planned. However, some policymakers and teachers cited a lack of quality assurance and monitoring tools to ascertain whether the programme was being implemented as planned.

*Because apart from just providing training on integrating CSE, there is need to monitor how the programme is being implemented to closely relate the programme outputs to the interventions. But you find that there is no criteria for monitoring and evaluating the programme impact, and it is difficult to assess the effectiveness of CSE* (Policymaker 2, Female, 39 yrs, IDI).

#### Insufficient time allocation for CSE

Most teachers recounted the lack of adequate time to teach CSE. This compromised the dose of CSE given to pupils. Invariably, teachers would only dedicate less than 5 min to teach CSE. This was compounded by the CSE not being incorporated into the school timetable and having been integrated into carrier subjects.

*I feel the impact of CSE is little because it is something that we teach in just 3–5 min; also, the programme is not on the timetable, and there is no curriculum to guide us on time and frequency of teaching* (Teacher, Female, 50yrs, IDI, school 1).

#### Lack of skills and competency to teach CSE

In all the participating schools, few teachers had received training in CSE. Document analysis indicated that CSE training was mostly done among guidance teachers. Some teachers recounted that the hesitancy to teach was due to a lack of skills and training on how to integrate CSE into other carrier subjects.

*Most teachers are not trained in CSE, and when asked to teach, they say that we are not trained in CSE, and hence we cannot teach (*Teacher, Male, 37, IDI, School 2).

### Mechanisms of CSE impact

Through qualitative interviews, we explored ways the implementation of CSE was perceived to have positively impacted the pupils regarding changes in sexual attitudes and acceptability of the CSE programme. This was based on feedback from pupils, teachers, and parents.

#### Feedback from pupils

Overall, qualitative data from pupils indicated that, despite the lack of consistency in CSE teaching and delivery methods such as role play and group discussions, most pupils found the programme interesting. Specifically, pupils cited enhancement of their SRH knowledge and skills and change in their SRH practices. Pupils also highlighted that the CSE programme helped them to freely discuss sexual issues with their teachers and parents.

*I have learnt from the programme [CSE] that I must change for the better because I now know the dangers that come with having multiple partnerships. I have also learned that I’m supposed to abstain now from sex. The programme is important and should be extended to all other schools where it has not been implemented* (Pupil, Female,17 yrs, IDI, School 7*).*

#### Feedback from teachers

Despite the negative perception of CSE by most teachers, some teachers indicated that the programme was very helpful in effecting SRH changes among pupils. Specifically, some teachers cited observable changes in pupil’s SRH attitudes, such as being able to report school and home-based gender-based violence to their guidance teachers.

*Despite many challenges surrounding the programme implementation, I have been able to see attitudinal and behavioural changes among pupils. For instance, pupils are now able to report gender-based violence cases happening at home and in schools, previously, it never used to happen* (Teacher, 16, Female, 46 yrs, IDI, School 9).

#### Feedback from parents

Some parents cited that the CSE programme had helped to enhance SRH knowledge and communication among pupils, which most parents could not easily do due to cultural taboos. Specifically, one female parent cited that her daughter was able to share with her what she had been taught during CSE lessons.

The *programme has been helpful to our children because they are now taught things about their sexuality which we fail to teach them due to cultural taboos. My daughter is now able to discuss things that they are taught at school regarding CSE. However, there is a need to make it culturally appropriate by including the divergent cultural views through stakeholder participants* (Parent 2, Female 48 yrs, IDI).

#### Fidelity, dose and reach

We assessed fidelity (whether CSE lessons were being delivered as per the curriculum), reach (how many pupils attended CSE lessons) and dose (whether CSE lessons were delivered in the planned time duration) through IDIs, document analysis and classroom observation.

#### Fidelity

The qualitative evaluation showed that CSE teaching was erratic in all the participating schools. Teachers cited a lack of guidance from the curriculum. Document analysis showed no inclusion of CSE in the school timetables and no indicators to measure programme activities and outcomes. Additionally, the CSE framework did not specify when and how to teach CSE. As such, how and when to teach CSE was at the discretion of teachers.

*It is difficult to teach because there is no timetable to guide us. Also, due to the busy schedule, we usually fail to integrate the programme. Teaching CSE is usually by chance whenever the teacher finds free time* (Teacher, Male, 34 yrs, IDI, School 9).

*Regarding monitoring and evaluation of the programme (CSE), it is difficult because there are no tools, mechanisms, and indicators to guide the process. The CSE framework has also not provided these*. (Policymaker, Female, 45yrs, IDI)

#### Dose

The frequency and duration of teaching varied among teachers. In many cases, teachers would only allocate five minutes to teaching CSE and as part of the introduction when teaching carrier subjects such as science, home economics and biology. Teachers attributed this to the busy school schedule and CSE not being included on the school timetable. Feedback from pupils indicated that pupils were not able to ask questions due to limited time. Also, teachers were not incorporating multiple delivery methods such as drama and role play.

*We don’t usually learn much because most teachers only spend about five minutes to teach. Most of the teachers usually say that they don’t have enough time to teach. Other teachers do not even teach it, we are usually not allowed to ask questions due to limited time* (Pupil, Female, 19 yrs, IDI, School 4).

*We are usually given 1 hour per period to teach. How do you then expect me to integrate CSE into a carrier subject in one hour? This whole thing [CSE integration] is not feasible. You will find that most teachers just use their first five minutes of teaching for CSE* (Teacher, Male, 39 yrs, IDI, School 7).

#### Reach

Document analysis indicated that the CSE programme was obligatory for all pupils and was endorsed by authorities from all participating schools, and all pupils were required to attend the CSE lessons. However, we observed that there was no mechanism to monitor whether all pupils were attending CSE lessons. Feedback from interviews with pupils indicated that most teachers were not consistent in teaching CSE. As such, some pupils thought that the programme was not a priority like other subjects.

*All pupils are required to learn CSE, but you will find that teachers are not consistent in teaching and therefore pupils do not take the programme seriously like other subjects. So, most pupils usually miss CSE lessons, and it is not a big issue for them, after all, the programme is not even examinable* (Pupil, Female, 18 yrs, IDI, School 5).

## Discussion

This study aimed to assess the quality of the CSE implementation, development process and contextual factors using recommendations of the MRC guidelines and the European Expert Group guidance on evaluating sexuality education programmes. Data revealed discrepancies between the Zambian CSE framework and the way CSE was implemented in schools. The compromise in the quality criteria of CSE implementation was due to lack of time and CSE prioritisation, omission of sensitive themes by teachers and non-inclusion of CSE on the school timetable. Despite many schools having implemented the CSE programme, findings revealed that there were no linkages between CSE and SRH services in the community. Linking CSE to SRH services in the community is an essential quality criteria in the implementation of CSE [11, 31, 35], and is critical to enhancing adolescents’ access to SRH services [42]. Ketting et al. [35], have noted that lack of establishing linkages between CSE and SRH services has the potential to compromise the quality of CSE implementation, because even though pupils may have adequate sexual health knowledge from CSE lessons, lack of access to SRH services could undermine the overall goal of CSE. In particular, linking CSE with SRH facilities  in communities is important because pupils need to know where they can access SRH services in the community [35]. To enhance collaboration and linkages for accessing SRH services by the pupils, most sexuality education curricula in European countries include class visits to SRH facilities [35]. However, this requires a multi-pronged approach with the involvement of key stakeholders such as healthcare providers to address barriers to accessing SRH services in health facilities [30].

CSE coupled with adequate CSE materials and comprehensive teacher training are critical to effective CSE integration and adaptation in schools [30, 55–57]. In light of this, the World Health Organization has set key competencies for sexuality educators, which include educator skills and competency [58]. This has implications for the quality of implementation of the CSE [59, 60], because untrained teachers are more likely to resist CSE, and they have negative attitudes and usually experience challenges utilising facilitative methods [61, 62]. Conversely, teachers trained in CSE better understand the dynamics of their class and are likely to address difficult relationships and cultural issues [63]. However, the challenge is inadequate funding towards CSE which constrains teacher training programmes [1]. Invariably, CSE funding is donor-supported and usually fragmented [64]. This has implications for the quality of CSE implementation and its sustainability beyond the funding period [1, 65]. As observed in Senegal, adequate funding to support CSE programmes from the central government is critical to developing teaching and learning materials, and sustainability of CSE in schools [66].

UNESCO guidelines on implementing sexuality education programmes stress emphasis on the positive approach to sexuality with the recognition of all sexual diversity and orientations [11]. However, owing to restrictive social-political environments CSE curricula in many LMICs are mostly heteronormative and stress emphasis on cisgender identities. In the current findings, we noted that though the CSE curriculum emphasised a positive approach to sexuality, the diversity of genders and sexual orientations were never emphasised during lessons. Hobaica et al. [67], have argued that this approach to teaching CSE marginalises sexual minorities such as homosexuals by not exploring their identities and promoting safe sexual practices. Similar findings have been reported in other settings [1, 9, 30, 68]. Contrary, trans-inclusive sexuality education can lead to increased normalisation, decreased gender dysphoria and positive health outcomes among pupils [67]. Research suggests that CSE curriculum needs to be gender sensitive to address gender norms shaped by cultural, social, and biological differences [69].

Social opposition to CSE has been linked to the non-involvement of stakeholders (parents, teachers and pupils) during the development and implementation of CSE [3, 1, 63, 70]. Stakeholder collaboration in the design and implementation of CSE aids in devising a culturally sensitive CSE [55, 63]. This collaboration entails working closely with community advisory boards, which could consist of relevant policymakers and interreligious and traditional leaders as demonstrated in Nigeria and Uganda [11, 30, 71]. The participatory approach to CSE enhances the co-creation of the CSE curriculum and provides a basis for understanding the lived experiences of key programme stakeholders, including challenges facing schools and how these influence teachers’ professional experiences [57, 72]. However, Ketting et al. [35], have argued that a participatory approach to CSE implementation could lead to a compromise in desired international quality standards of CSE. In addition, there is a challenge in reaching consensus because different stakeholders may have different beliefs and values [68]. Still, contextualised and culturally adapted CSE programmes offer more positive outcomes in terms of acceptability and addressing the socio-cultural needs of pupils, teachers, and parents [1]. Not only is this critical to the effective adaptation of the CSE programme, but it also provides for understanding the triadic reciprocal predictors of HIV and risky sexual behaviours among adolescents [73].

Broad contextual factors that could facilitate or hinder CSE implementation include sociocultural factors (religious and cultural beliefs, social norms) political factors (criminalisation of other sexual orientations such as homosexuality) and economic factors (lack of CSE resources and materials in schools) [1, 3, 9, 35, 59, 63]. In the current study, teachers’ personal biases, and opinions influenced by cultural beliefs and norms compromised the quality of CSE implementation. We noted that some teachers were holding back or skipping culturally sensitive topics while stressing emphasis on abstinence-only messages. Findings from this study support evidence from Ethiopia [55], Uganda [22], Kenya [23], Lesotho [74] and South Africa [75] where teachers recognised tensions between their cultural beliefs and curriculum content. Invariably, teachers tend to discuss culturally sensitive topics such as homosexuality in a negative light and adapt the content of CSE to conform to local norms in ways that undermine the goals and objectives of CSE [76]. Marielle et al. [57], have linked this to the interplay between teacher’s beliefs and practices and how they recontextualise the CSE policy in the wider socio-economic context. Teachers play a significant role in shaping the recontexualisation of CSE policy in schools. As such, CSE programme development should include teachers’ views, concerns and daily realities to help them reconcile personal religious and cultural beliefs and some of the content of the CSE [77]. Contrary, findings also show that there was a high acceptability of the CSE programme among pupils. Facilitators of CSE implementation in schools were related to the increase in pupils’ SRH knowledge and perceived changes in practices such as abstaining from sexual intercourse and reporting sexual and gender-based violence. However, emphasis on abstinence is a pronounced criticism of CSE programmes in many LMICs because these programmes provide incomplete information and are often neglectful to sexually active adolescents [22, 55].

Enhancing programme fidelity is essential for programme outcomes to be highly attributed to the intervention and provides a basis for understanding how and why an intervention works [78, 79]. In the current study, teacher characteristics, lack of guidance from the curriculum on the time and frequency of teaching, lack of teacher training in CSE and limited time allocation to teaching CSE affected CSE programme adherence, dose and the quality of the lessons given. This finding corroborates with findings reported elsewhere [80]. Training in CSE is essential to enable teachers to understand the purpose and content of the curriculum and to competently cover key CSE concepts [79]. Lachausse et al. [80], also recommend factors such as strong support for administration, characteristics of the curriculum and provision of ongoing technical assistance to increase implementation fidelity [80]. Apart from challenging existing cultural norms and beliefs, the use of training to support teachers, the use of technical assistance, providing tools to teachers such as manuals and guides and engaging in continuous quality improvement could be crucial to enhance fidelity in CSE programme implementation [79].

Monitoring and evaluation form an essential component of improving the quality of the implementation process of the CSE programme [11]. Monitoring and evaluation of CSE implementation is critical in providing stakeholders with programme information to enhance responsiveness. Data in the current study showed that effective implementation was affected by a lack of monitoring tools and activities at all the participating schools. Similar findings have been reported in Kenya [1]. Barden et al. [81], have recommended essential monitoring and evaluation practices for sexuality education, which include continuous stakeholder engagement, development of indicators to measure programme activities and outcomes and collection and use of data to calculate the indicators.

## Conclusion

The complex influences of contextual factors during CSE implementation highlight the need for contextual analysis during the interventional design. Co-creation of the CSE programme through stakeholder participation could reduce social opposition and enable the development of a culturally sensitive CSE. Additionally, comprehensive teacher training, a guiding curriculum as well as establishment of appropriate monitoring tools and indicators could be vital in enhancing the quality of implementation of CSE.

### Limitations

The study was not without limitations, owing to the sensitivity of the topic, some pupils and teachers might have provided biased and inaccurate responses. However, triangulating the data collection sources was essential in ensuring the credibility of the data. Also, independent researchers conducted the evaluation, further reducing the probability of bias in interpreting the evaluation results. To minimise bias associated with reporting the process evaluation, international evaluation guidelines were observed.

## Data Availability

The data used and analysed during the current study are available from the corresponding author upon reasonable request.

## Abbreviations

HIV: Human immunodeficiency virus
SRH: Sexual and reproductive health
IDI: In-depth interview
FGD: Focus group discussion
CSE: Comprehensive sexuality education
MRC: Medical research council

## References

[1] Keogh SC, Stillman M, Awusabo-Asare K, Sidze E, Monzón AS, Motta A, et al. Challenges to implementing national comprehensive sexuality education curricula in low- and middle-income countries: case studies of Ghana, Kenya, Peru and Guatemala. PLoS One. 2018;13(7):e0200513. 10.1371/journal.pone.0200513. 10.1371/journal.pone.0200513 PMC6040779 29995942

[2] Keogh SC, Leong E, Motta A, Sidze E, Monzón AS, Amo-Adjei J. Classroom implementation of national sexuality education curricula in four low- and middle-income countries. Sex Educ. 2020. 10.1080/14681811.2020.1821180. 10.1080/14681811.2020.1821180

[3] Zulu JM, Blystad A, Haaland MES, Michelo C, Haukanes H, Moland KM. Why teach sexuality education in school? Teacher discretion in implementing comprehensive sexuality education in rural Zambia. Int J Equity Health. 2019;18(1):116. 10.1186/s12939-019-1023-1. 31558168 10.1186/s12939-019-1023-1 PMC6764121

[4] Goldman JDG. UNESCO’s guidance on puberty and sexual health education for students aged 9–12 years compared to an upper primary school curriculum. Health Educ J. 2015. 10.1177/0017896914537004. 10.1177/0017896914537004

[5] UNESCO. No TitleZambia | Comprehensive sexuality education | Education profiles. 2023. Available from: https://education-profiles.org/sub-saharan-africa/zambia/~comprehensive-sexuality-education.

[6] Abdurahman C, Oljira L, Hailu S, Mengesha MM. Sexual and reproductive health services utilization and associated factors among adolescents attending secondary schools. Reprod Health. 2022;19(1):1–10. Available from: (https://reproductive-health-journal.biomedcentral.com/articles/10.1186/s12978-022-01468-w . Cited 2024 Jan 25 ). 35840973 10.1186/s12978-022-01468-w PMC9287868

[7] Mbarushimana V, Goldstein S, Conco DN. "Not just the consequences, but also the pleasurable sex": a review of the content of comprehensive sexuality education for early adolescents in Rwandae. BMC Public Health. 2023;23(49):14. 10.21203/rs.3.rs-1784765/v1. Available from (https://www.ncbi.nlm.nih.gov/pmc/articles/PMC9824976/pdf/12889_2022_Article_14966.pdf). 10.1186/s12889-022-14966-0 PMC9824976 36609366

[8] Kemigisha E, Bruce K, Ivanova O, Leye E, Coene G, Ruzaaza GN, Ninsiima AB, Mlahagwa W, Nyakato VN, Michielsen K. Evaluation of a school based comprehensive sexuality education program among very young adolescents in rural Uganda. BMC Public Health. 2019;19(1):1–11. Available from(https://bmcpublichealth.biomedcentral.com/articles/10.1186/s12889-019-7805-y . Cited 2023 Sep 9 ). 31660918 10.1186/s12889-019-7805-y PMC6819440

[9] Chavula MP, Zulu JM, Hurtig AK. Factors influencing the integration of comprehensive sexuality education into educational systems in low- and middle-income countries: a systematic review. Reprod Health. 2022;19(1). 10.1186/s12978-022-01504-9. Available from: https://pubmed.ncbi.nlm.nih.gov/36175901/. Cited 2022 Dec 10. 10.1186/s12978-022-01504-9 PMC9524136 36175901

[10] Yakubu I, Salisu WJ. Determinants of adolescent pregnancy in sub-Saharan Africa: a systematic review. Reprod Health. 2018;15(1):1–11. Available from (https://reproductive-health-journal.biomedcentral.com/articles/10.1186/s12978-018-0460-4 . Cited 2024 Jan 25 ). 29374479 10.1186/s12978-018-0460-4 PMC5787272

[11] Herat J, Plesons M, Castle C, Babb J, Chandra-Mouli V. The revised international technical guidance on sexuality education - a powerful tool at an important crossroads for sexuality education. Reprod Health. 2018;15(1):1–4 (Available from: https://reproductive-health-journal.biomedcentral.com/articles/10.1186/s12978-018-0629-x. Cited 2024 Jan 25). 30400902 10.1186/s12978-018-0629-x PMC6220458

[12] Mabaso M, Maseko G, Sewpaul R, Naidoo I, Jooste S, Takatshana S, Reddy T, Zuma K, Zungu N. Trends and correlates of HIV prevalence among adolescents in South Africa: evidence from the 2008, 2012 and 2017 South African National HIV prevalence, incidence and behaviour surveys. AIDS Res Ther. 2021;18(1):1–8 (Available from: https://aidsrestherapy.biomedcentral.com/articles/10.1186/s12981-021-00422-3. Cited 2023 Sep 9 ). 34906170 10.1186/s12981-021-00422-3 PMC8670218

[13] Hunter SC, Russell K, Pagani S, Munro L, Pimenta SM, Marín-López I, Hong JS, Knifton L. A social-ecological approach to understanding adolescent sexting behavior. Arch Sex Behav. 2021;50(6):2347–57 (Available from: https://link.springer.com/article/10.1007/s10508-021-01988-9. Cited 2023 Sep 9 ). 33982213 10.1007/s10508-021-01988-9 PMC8416823

[14] McCarraher DR, Packer C, Mercer S, Dennis A, Banda H, Nyambe N, et al. Adolescents living with HIV in the copperbelt province of Zambia: their reproductive health needs and experiences. PLoS One. 2018. 10.1371/journal.pone.0197853. 29870562 10.1371/journal.pone.0197853 PMC5988282

[15] Ndagijimana E, Biracyaza E, Nzayirambaho M. Risky sexual behaviors and their associated factors within high school students from Collège Saint André in Kigali, Rwanda: an institution-based cross-sectional study. Front Reprod Heal. 2023;5:10 (Available from: https://www.frontiersin.org/articles/10.3389/frph.2023.1029465/full ). 10.3389/frph.2023.1029465 PMC10020213 36936133

[16] Bukuluki PMW, Kisaakye P, Wandiembe SP, Kiwujja V, Kajungu C, Mugwanya W, et al. Utilization of sexual and reproductive health services among young people in refugee settings in Uganda. Front Reprod Heal. 2023;5. 10.3389/frph.2023.1077761. 10.3389/frph.2023.1077761 PMC9998478 36910338

[17] Comprehensive sexuality education in Zambia | UNAIDS. Available from: https://www.unaids.org/en/resources/presscentre/featurestories/2016/november/20161109_zambia. Cited 2023 Sep 9.

[18] Parry H, Wilentz G. Emerging Evidence, Lessons and Practice in Comprehensive Sexuality Education: A global review. 2015. Available from: https://resourcecentre.savethechildren.net/document/emerging-evidence-lessons-and-practice-comprehensive-sexuality-education-global- .

[19] Gelehkolaee KS, Azin SA, Nedjat S, Hajiabadi AZ, Maasoumi R. Reaching consensus: a scoping review on school-based comprehensive sexuality education programs (CSE). Práxis Educ. 2020;16(37). 10.22481/praxisedu.v16i37.6175. Available from: https://periodicos2.uesb.br/index.php/praxis/article/view/6175.

[20] Haberland NA. The case for addressing gender and power in sexuality and HIV education: a comprehensive review of evaluation studies. Int Perspect Sex Reprod Health. 2015;41(1):31–42. 10.1363/4103115. Available from: https://pubmed.ncbi.nlm.nih.gov/25856235/. Cited 2024 Mar 27. 10.1363/4103115 25856235

[21] Achen D, Fernandes D, Kemigisha E, Rukundo GZ, Nyakato VN, Coene G. Trends and challenges in Comprehensive Sex Education (CSE) research in Sub-Saharan Africa: a narrative review. Curr Sex Heal Rep. 2023;1–9. 10.1007/s11930-023-00362-1. Available from: http://www.ncbi.nlm.nih.gov/pubmed/37362203. Cited 2023 Dec 15. 10.1007/s11930-023-00362-1 PMC10163565 37362203

[22] Vanwesenbeeck I, Westeneng J, de Boer T, Reinders J, van Zorge R. Lessons learned from a decade implementing comprehensive sexuality education in resource poor settings: the world starts with me. Sex Educ. 2016;16(5):471–86. 10.1080/14681811.2015.1111203. 10.1080/14681811.2015.1111203

[23] Sidze EM, Stillman M, Keogh S, Mulupi S, Egesa CP, Leong E, Mutua M, Muga W, Bankole A, Izugbara CO. From paper to practice: sexuality education policies and their implementation in Kenya. Guttmacher Institute. https://www.guttmacher.org/sites/default/files/report_pdf/sexuality-education-kenya-report.pdf.

[24] Kangaude G, Coast E, Fetters T. Adolescent sexual and reproductive health and universal health coverage: a comparative policy and legal analysis of Ethiopia, Malawi and Zambia. Sex Reprod Heal Matters. 2020;28(2). 10.1080/26410397.2020.183229. Available from: https://www.ncbi.nlm.nih.gov/pmc/articles/PMC7887923/. Cited 2024 Jan 25. 10.1080/26410397.2020.1832291 PMC7887923 33121392

[25] Moore GF, Audrey S, Barker M, Bond L, Bonell C, Hardeman W, et al. Process evaluation of complex interventions: medical research council guidance. 10.1136/bmj.h1258. Available from: http://www.bmj.com/. Cited 2023 Sep 8. 10.1136/bmj.h1258 PMC4366184 25791983

[26] Chandra-Mouli V, Svanemyr J, Amin A, Fogstad H, Say L, Girard F, et al. Twenty years after international conference on population and development: where are we with adolescent sexual and reproductive health and rights? J Adolesc Heal. 2015;56(1):S1–6. 10.1016/j.jadohealth.2014.09.015. 10.1016/j.jadohealth.2014.09.015 25528975

[27] Grant A, Treweek S, Dreischulte T, Foy R, Guthrie B. Process evaluations for cluster-randomised trials of complex interventions: a proposed framework for design and reporting. Trials. 2013;14(1):1–10. 10.1186/1745-6215-14-15. Available from: https://trialsjournal.biomedcentral.com/articles/10.1186/1745-6215-14-15. Cited 2023 Sep 9. 10.1186/1745-6215-14-15 PMC3600672 23311722

[28] Mukoma W, Flisher AJ, Ahmed N, Jansen S, Mathews C, Klepp KI, et al. Process evaluation of a school-based HIV/AIDS intervention in South Africa. Scand J Public Health. 2009;37 Suppl 2(SUPPL. 2):37–47. 10.1177/140349480809063. Available from: https://pubmed.ncbi.nlm.nih.gov/19493980/. 10.1177/1403494808090631 19493980

[29] Paulsen MM, Varsi C, Andersen LF. Process evaluation of the implementation of a decision support system to prevent and treat disease-related malnutrition in a hospital setting. BMC Health Serv Res. 2021;21(1). 10.1186/s12913-021-06236-3. https://www.ncbi.nlm.nih.gov/pmc/articles/PMC7995565/. Cited 2023 Sep 9. 10.1186/s12913-021-06236-3 PMC7995565 33766017

[30] Kemigisha E, Ivanova O, Ruzaaza GN, Ninsiima AB, Kaziga R, Bruce K, et al. Process evaluation of a comprehensive sexuality education intervention in primary schools in South Western Uganda. Sex Reprod Healthc. 2019;21:51–9. 10.1016/j.srhc.2019.06.006. Available from: https://pubmed.ncbi.nlm.nih.gov/31395234/. Cited 2023 Sep 13. 10.1016/j.srhc.2019.06.006 31395234

[31] Chandra-Mouli V, Lane C, Wong S. What does not work in adolescent sexual and reproductive health: a review of evidence on interventions commonly accepted as best practices. Glob Heal Sci Pract. 2015;3(3):333. 10.9745/GHSP-D-15-00126. Available from: https://www.ncbi.nlm.nih.gov/pmc/articles/PMC4570008/. Cited 2024 Jan 25. 10.9745/GHSP-D-15-00126 PMC4570008 26374795

[32] Michielsen K, Chersich MF, Luchters S, De Koker P, Van Rossem R, Temmerman M. Effectiveness of HIV prevention for youth in sub-Saharan Africa: systematic review and meta-analysis of randomized and nonrandomized trials. AIDS. 2010;24(8):1193–202. 10.1097/QAD.0b013e3283384791. Available from: https://pubmed.ncbi.nlm.nih.gov/20375876/. Cited 2024 Jan 25. 10.1097/QAD.0b013e3283384791 20375876

[33] Ivanova O, Rai M, Michielsen KDS. How sexuality education programs have been evaluated in low- and lower-middle-income countries? A systematic review. Int J Env Res Public Heal. 2020;17(21):8183. 10.3390/ijerph17218183. 10.3390/ijerph17218183 PMC7664002 33167481

[34] Fernandes D, Kemigisha E, Achen D, et al. Process evaluation of a parent-child communication intervention for adolescent sexual and reproductive health in Uganda. BMC Public Health. 2024;24(319). Available from: https://bmcpublichealth.biomedcentral.com/articles/10.1186/s12889-023-17513-7#citeas. 10.1186/s12889-023-17513-7 PMC10826092 38287314

[35] Ketting E, Friele M, Michielsen K. Evaluation of holistic sexuality education: a European expert group consensus agreement. Eur J Contracept Reprod Health Care. 2016;21(1):68–80. 10.3109/13625187.2015.1050715. Available from: https://pubmed.ncbi.nlm.nih.gov/26024010/. Cited 2023 Sep 9. 10.3109/13625187.2015.1050715 26024010

[36] Country progress report-Zambia. Country progress report-Zambia. Global AIDS monitoring 2019. Available from https://www.unaids.org/sites/default/files/country/documents/ZMB_2019_countryreport.pdf. Cited on Sep 10.

[37] Malunga G, Sangong S, Saah FI, Bain LE. Prevalence and factors associated with adolescent pregnancies in Zambia: a systematic review from 2000–2022. Arch Public Heal. 2023;81(27). Available from: https://archpublichealth.biomedcentral.com/articles/10.1186/s13690-023-01045-y#citeas. 10.1186/s13690-023-01045-y PMC9940412 36805786

[38] Simooya C, Silumbwe A, Halwindi H, Zulu JM, Nzala S. Exploring communication and implementation challenges of the HIV/AIDS policy change to test-and-treat-all in selected public health facilities in Lusaka District, Zambia. Implement Sci Commun. 2023;4(1). 10.1186/s43058-023-00430-6. https://www.ncbi.nlm.nih.gov/pmc/articles/PMC10176665/. 10.1186/s43058-023-00430-6 PMC10176665 37173757

[39] Chavula MP, Zulu JM, Goicolea I, Hurtig AK. Unlocking policy synergies, challenges and contradictions influencing implementation of the comprehensive sexuality education framework in Zambia: a policy analysis. Heal Res Policy Syst. 2023;21(1):1–15 (Available from: https://health-policy-systems.biomedcentral.com/articles/10.1186/s12961-023-01037-y. Cited 2024 Jan 25 ). 10.1186/s12961-023-01037-y PMC10500755 37710251

[40] Wekesah FM, Nyakangi V, Onguss M, Njagi J, Bangha M. Comprehensive sexuality education in sub-Saharan Africa. African Popul Heal Res Cent (APHRC); 2019. https://aphrc.org/wp-content/uploads/2019/12/COMPREHENSIVE-SEXUALITY-EDUCATION-IN-SUB-SAHARAN-AFRICA-1.pdf.

[41] Rogers E, Hemal K, Tembo Z, Mukanu M, Mbizvo M, Bellows B. Comprehensive sexuality education for adolescents in Zambia via the mobile-optimized website TuneMe: a content analysis. Am J Sex Educ. 2020;15(1):82. 10.1080/15546128.2019.1635546. 10.1080/15546128.2019.1635546

[42] Mbizvo MT, Kasonda K, Muntalima NC, Rosen JG, Inambwae S, Namukonda ES, Mungoni R, Okpara N, Phiri C, Chelwa N, Kangale C. Comprehensive sexuality education linked to sexual and reproductive health services reduces early and unintended pregnancies among in-school adolescent girls in Zambia. BMC Public Health. 2023;23(1):1–13 (Available from: https://bmcpublichealth.biomedcentral.com/articles/10.1186/s12889-023-15023-0. Cited 2023 Sep 13 ). 36797703 10.1186/s12889-023-15023-0 PMC9933327

[43] Chirwa-Kambole E, Svanemyr J, Sandøy I, Hangoma P, Zulu JM. Acceptability of youth clubs focusing on comprehensive sexual and reproductive health education in rural Zambian schools: a case of Central Province. BMC Health Serv Res. 2020;20(1):42. 10.1186/s12913-020-4889-0. 31948452 10.1186/s12913-020-4889-0 PMC6966797

[44] Samadaee Gelehkolaee K, Maasoumi R, Azin SA, Nedjat S, Parto M, Zamani Hajiabadi I. Stakeholders’ perspectives of comprehensive sexuality education in Iranian male adolescences. Reprod Health. 2021;18(26):13 (Available from: https://reproductive-health-journal.biomedcentral.com/articles/10.1186/s12978-021-01084-0 ). 33618726 10.1186/s12978-021-01084-0 PMC7901096

[45] Zambia Statistical Agency. Zambia population-based HIV impact assessment 2021. 2021. Available from: https://www.zamstats.gov.zm/wp-content/uploads/2023/12/ZAMPHIA-2021-Final-Report-December-2023.pdf . Cited on Sep 30.

[46] Mwanza J, Kawonga M, Kumwenda A, Gray GE, Mutale W, Doherty T. Health system response to preventing mother-to-child transmission of HIV policy changes in Zambia: a health system dynamics analysis of primary health care facilities. Glob Health Action. 2022;15(1). 10.1080/16549716.2022.2126269. Available from: https://www.ncbi.nlm.nih.gov/pmc/articles/PMC9578454/. Cited 2024 Jan 26. 10.1080/16549716.2022.2126269 PMC9578454 36239946

[47] Kitwe (District, Zambia) - population statistics, charts, map and location. Available from: https://www.citypopulation.de/en/zambia/wards/admin/0204__kitwe/. Cited 2023 Sep 13.

[48] Parmley LE, Nkumbula T, Chilukutu L, Chelu L, Mulemfwe C, Hanunka B, et al. Impacts of COVID-19 on sexual risk behaviors, safe injection practices, and access to HIV services among key populations in Zambia: findings from a rapid qualitative formative assessment. PLoS One. 2023;18(8). Available from: https://journals.plos.org/plosone/article?doi=10.1371/journal.pone.0289007. 10.1371/journal.pone.0289007 PMC10393175 37527283

[49] (USAID) USA for ID, Doors O. Understanding the legal barriers to accessing HIV/AIDS services by key populations: findings from expert panel meetings in Zambia. 2017. Available from: https://www.fhi360.org/sites/default/files/media/documents/resource-zambia-open-doors-expert-panel-report.pdf.

[50] Palinkas LA, Horwitz SM, Green CA, Wisdom JP, Duan N, Hoagwood K. Purposeful sampling for qualitative data collection and analysis in mixed method implementation research. Adm Policy Ment Heal Ment Heal Serv Res. 2015;42(5):533–44 (Available from: https://link.springer.com/article/10.1007/s10488-013-0528-y. Cited 2023 Sep 9 ). 10.1007/s10488-013-0528-y PMC4012002 24193818

[51] UNESCO. International technical guidance on sexuality education: an evidence-informed approach. 2018. https://unesdoc.unesco.org/ark:/48223/pf0000260770.

[52] Bingham AJ. From data management to actionable findings: a five-phase process of qualitative data analysis. Int J Qual Methods. 2023;22:1–11 (Available from: https://journals.sagepub.com/doi/full/10.1177/16094069231183620. Cited 2023 Sep 9 ). 10.1177/16094069231183620

[53] Nowell LS, Norris JM, White DE, Moules N. Thematic analysis: striving to meet the trustworthiness criteria. Int J Qual Methods. 2017;16:1–13 (Available from: https://journals.sagepub.com/doi/10.1177/1609406917733847 ). 10.1177/1609406917733847

[54] Carter N, Bryant-Lukosius D, Dicenso A, Blythe J, Neville AJ. The use of triangulation in qualitative research. Oncol Nurs Forum. 2014;41(5):545–7. 10.1188/14.ONF.545-547. Available from: https://pubmed.ncbi.nlm.nih.gov/25158659/. Cited 2023 Sep 9. 10.1188/14.ONF.545-547 25158659

[55] Browes NC. Comprehensive sexuality education, culture and gender: the effect of the cultural setting on a sexuality education programme in Ethiopia. Sex Educ. 2015;15(6):655–70. 10.1080/14681811.2015.1065476. 10.1080/14681811.2015.1065476

[56] de Haas B, Hutter I. Teachers’ professional identities in the context of school-based sexuality education in Uganda—a qualitative study. Health Educ Res. 2020;35(6):553–63. 10.1093/her/cyaa044. (Cited 2023 Sep 13). 33367770 10.1093/her/cyaa044 PMC7768668

[57] Le Mat MLJ, Miedema EAJ, Kosar-Altinyelken H. Moulding the teacher: factors shaping teacher enactment of comprehensive sexuality education policy in Ethiopia. J Comparat Int Educ. 2019;51(6):862–80. 10.1080/03057925.2019.1682967. 10.1080/03057925.2019.1682967

[58] World Health Organization. Training matters: a framework for core competencies of sexuality educators. 2023:1.8.11.17, pg 1–64. https://www.educationsexuelle-ecole.ch/ck/ckfinder/userfiles/files/BZgA-training-framework.pdf.

[59] Mercelline A, Ogolla MO. Assessment of the implementation of comprehensive sexuality education in Kenya. Afr J Reprod Heal. 2019;23(2):110–20. 10.29063/ajrh2019/v23i2.11. Available from: https://pubmed.ncbi.nlm.nih.gov/31433599/. 10.29063/ajrh2019/v23i2.11 31433599

[60] Leung H, Shek DTL, Leung E, Shek EYW. Development of contextually-relevant sexuality education: lessons from a comprehensive review of adolescent sexuality education across cultures. Int J Environ Res Public Health. 2019;16(4). 10.3390/ijerph16040621. Available from: https://www.ncbi.nlm.nih.gov/pmc/articles/PMC6406865/. Cited 2023 Sep 13. 10.3390/ijerph16040621 PMC6406865 30791604

[61] Emambokus WBS, Oogarah-Pratap B. Exploring parents’ and teachers’ perspectives about school-based sexuality education in a multicultural context: a case study in Mauritius. Educ Process Int J. 2019;8(3):185–95. 10.22521/edupij.2019.83.3. 10.22521/edupij.2019.83.3

[62] Panchaud C, Keogh SC, Stillman M, Awusabo-Asare K, Motta A, Sidze E, Monzón AS. Towards comprehensive sexuality education: a comparative analysis of the policy environment surrounding school-based sexuality education in Ghana, Peru, Kenya and Guatemala. Sex Educ. 2019;9(3):277–96 (Available from: https://www.tandfonline.com/doi/epdf/10.1080/14681811.2018.1533460?src=getftr ). 10.1080/14681811.2018.1533460

[63] Chavula MP, Svanemyr J, Zulu JM, Sandøy IF. Experiences of teachers and community health workers implementing sexuality and life skills education in youth clubs in Zambia. Glob Public Health. 2022;17(6):926–40. 10.1080/17441692.2021.1893371. Available from: https://pubmed.ncbi.nlm.nih.gov/33661081/. Cited 2022 Dec 10. 10.1080/17441692.2021.1893371 33661081

[64] Wangamati CK. Comprehensive sexuality education in sub-Saharan Africa: adaptation and implementation challenges in universal access for children and adolescents. Sex Reprod Heal Matters. 2020;28(2). Available from: https://click.endnote.com/viewer?doi=10.1080%2F26410397.2020.1851346. Cited 2022 Dec 10. 10.1080/26410397.2020.1851346 PMC7887764 33295853

[65] Wooda L, Rollerib LA. Designing an effective sexuality education curriculum for schools: lessons gleaned from the South(ern) African literature. Sex Educ Sex Soc. 2014;14(5):525–42. Available from (https://www.tandfonline.com/doi/abs/10.1080/14681811.2014.918540). 10.1080/14681811.2014.918540

[66] Chau K, Traoré Seck A, Chandra-Mouli V, Svanemyr J. Scaling up sexuality education in Senegal: integrating family life education into the national curriculum. Sex Educ. 2016. 10.1080/14681811.2015.1123148. https://www.sexrightsafrica.net/wp-content/uploads/2016/07/Senegal-CSE-Case-Study.pdf.

[67] Hobaica S, Schofield K, Kwon P. Here’s your anatomy?Good luck”: Transgender individuals in cisnormative sex education. Am J Sex Educ. 2019;14(3):358–87 (Available from: https://www.tandfonline.com/doi/abs/10.1080/15546128.2019.1585308 ).

[68] Wangamati CK. Comprehensive sexuality education in sub-Saharan Africa: adaptation and implementation challenges in universal access for children and adolescents. Sex Reprod Heal Matters. 2020;28(2). 10.1080/26410397.2020.1851346. Available from: https://www.tandfonline.com/action/journalInformation?journalCode=zrhm21. Cited 2023 Sep 14. 10.1080/26410397.2020.1851346 PMC7887764 33295853

[69] Herat J, Plesons M, Castle C, Babb J, Chandra-Mouli V. The revised international technical guidance on sexuality education - a powerful tool at an important crossroads for sexuality education. Reprod Health. 2018;15(1):1–4. Available from(https://reproductive-health-journal.biomedcentral.com/articles/10.1186/s12978-018-0629-x. Cited 2024 Mar 9 ). 30400902 10.1186/s12978-018-0629-x PMC6220458

[70] Mwape J, Munsaka E. Local-stakeholders’ engagement in the implementation of comprehensive sexuality education in selected public schools in Samfya District, Zambia. Int J Res Innov Soc Sci. 2020;6(2). Available from: https://www.rsisinternational.org/journals/ijriss/Digital-Library/volume-6-issue-2/01-06.pdf.

[71] Huaynoca S, Chandra-Mouli V, Yaqub N, Denno DM. Scaling up comprehensive sexuality education in Nigeria: from national policy to nationwide application. Sex Educ. 2014;14(2):191–209. 10.1080/14681811.2013.856292. 10.1080/14681811.2013.856292

[72] Kirby DB, Laris BA, Rolleri LA. Sex and HIV education programs: their impact on sexual behaviors of young people throughout the world. J Adolesc Health. 2007;40(3):206–17. 10.1016/j.jadohealth.2006.11.143. Available from: https://pubmed.ncbi.nlm.nih.gov/17321420/. Cited 2023 Sep 13. 10.1016/j.jadohealth.2006.11.143 17321420

[73] Dos Santos LM. Promoting safer sexual behaviours by employing social cognitive theory among gay university students: a pilot study of a peer modelling programme. 2020. Int J Environ Res Public Health. 2020;17(5):1804. 10.3390/ijerph17051804. www.mdpi.com/journal/ijerph. Cited 2023 Sep 25. 10.3390/ijerph17051804 PMC7084672 32164309

[74] Khau M. “Our culture does not allow that”: exploring the challenges of sexuality education in rural communities. Perspect Educ. 2012;30(1):61–9. http://hdl.handle.net/11660/2846. Cited 2023 Nov 10.

[75] Helleve A, Flisher AJ, Onya H, Mukoma WKK. South African teachers’ reflections on the impact of culture on their teaching of sexuality and HIV/AIDS. Cult Heal Sex. 2009;11(2):189–204. 10.1080/13691050802562613. 10.1080/13691050802562613 19132582

[76] Francis DA. Sexuality education in South Africa. Int J Educ Dev. 2010;30(3):314–9. 10.1016/j.ijedudev.2009.12.003. 10.1016/j.ijedudev.2009.12.003

[77] Le Mat ML, Miedema EA, Amentie SA, Kosar-Altinyelken H. Moulding the teacher: factors shaping teacher enactment of comprehensive sexuality education policy in Ethiopia. Comp A J Comp Int Educ. 2019. 10.1080/03057925.2019.1682967. 10.1080/03057925.2019.1682967

[78] Carroll C, Patterson M, Wood S, Booth A, Rick J, Balain S. A conceptual framework for implementation fidelity. Implement Sci. 2007;2(40). Available from: https://implementationscience.biomedcentral.com/articles/10.1186/1748-5908-2-40#citeas. 10.1186/1748-5908-2-40 PMC2213686 18053122

[79] Barbee AP, Antle B, Langley C, Cunningham MR, Whiteside D, Sar BK, Archuleta A, Karam E, Borders K. How to ensure fidelity in implementing an evidence based teen pregnancy prevention curriculum. Child Youth Serv Rev. 2021;129:106175. 10.1016/j.childyouth.2021.106175

[80] Robert G, LaChausse KR, Clark SC. Beyond teacher training: the critical role of professional development in maintaining curriculum fidelity. J Adolesc Heal. 2014;54(3):S53–58. 10.1016/j.jadohealth.2013.12.029. Available from https://www.sciencedirect.com/science/article/pii/S1054139X13008574. 10.1016/j.jadohealth.2013.12.029 24560077

[81] Barden-O’Fallon J, McCallum E. Monitoring and evaluation of sexual and reproductive health programs. Glob Public Health. 2022. Available from: https://oxfordre.com/publichealth/display/10.1093/acrefore/9780190632366.001.0001/acrefore-9780190632366-e-213.

[82] World Medical Association Declaration of Helsinki: ethical principles for medical research involving human subjects. JAMA. 2013;310(20):2191–4. Available from: https://pubmed.ncbi.nlm.nih.gov/24141714/. Cited 2023 Oct 25. 10.1001/jama.2013.281053 24141714

